# VPsero: Rapid Serotyping of *Vibrio parahaemolyticus* Using Serogroup-Specific Genes Based on Whole-Genome Sequencing Data

**DOI:** 10.3389/fmicb.2021.620224

**Published:** 2021-09-02

**Authors:** Shengzhe Bian, Yangyang Jia, Qiuyao Zhan, Nai-Kei Wong, Qinghua Hu, Wenwei Zhang, Yongwei Zhang, Liqiang Li

**Affiliations:** ^1^BGI-Shenzhen, Shenzhen, China; ^2^BGI Education Center, University of Chinese Academy of Sciences, Shenzhen, China; ^3^School of Public Health (Shenzhen), Sun Yat-sen University, Guangzhou, China; ^4^Shenzhen Key Laboratory of Unknown Pathogen Identification, Shenzhen, China; ^5^CAS Key Laboratory of Tropical Marine Bio-resources and Ecology, South China Sea Institute of Oceanology, Chinese Academy of Sciences, Guangzhou, China; ^6^Department of Pharmacology, Shantou University Medical College, Shantou, China; ^7^Shenzhen Center for Disease Control and Prevention, Shenzhen, China

**Keywords:** *Vibrio parahaemolyticus*, lipopolysaccharide (LPS), capsular polysaccharide (CPS), genetic cluster, serotype

## Abstract

*Vibrio parahaemolyticus* has emerged as a significant enteropathogen in human and marine habitats worldwide, notably in regions where aquaculture products constitute a major nutritional source. It is a growing cause of diseases including gastroenteritis, wound infections, and septicemia. Serotyping assays use commercially available antisera to identify *V. parahaemolyticus* strains, but this approach is limited by high costs, complicated procedures, cross-immunoreactivity, and often subjective interpretation. By leveraging high-throughput sequencing technologies, we developed an *in silico* method based on comparison of gene clusters for lipopolysaccharide (LPSgc) and capsular polysaccharide (CPSgc) by firstly using the unique-gene strategy. The algorithm, VPsero, which exploits serogroup-specific genes as markers, covers 43 K and all 12 O serogroups in serotyping assays. VPsero is capable of predicting serotypes from assembled draft genomes, outputting LPSgc/CPSgc sequences, and recognizing possible novel serogroups or populations. Our tool displays high specificity and sensitivity in prediction toward *V. parahaemolyticus* strains, with an average sensitivity in serogroup prediction of 0.910 for O and 0.961 for K serogroups and a corresponding average specificity of 0.990 for O and 0.998 for K serogroups.

## Introduction

Serotyping is fundamentally important to disease control processes based on epidemiological surveys and identification of pathogenic signatures. Conventional serotyping methods employed in laboratories require considerable amounts of specialized reagents and expertise. For many bacterial pathogens, antigen diversity of structural lipopolysaccharide (LPS) and/or capsular polysaccharide (CPS) is a practical basis for serotyping and is controlled by the polysaccharide biosynthesis loci, principally gene clusters for LPS (LPSgc) and gene clusters for CPS (CPSgc), that encode proteins transporting monosaccharides and synthesizing polysaccharides. PCR-based genotyping targeting these polysaccharide biosynthesis loci is becoming prevalent, but it remains limited by genetic mutations of targeted genes. It is thus primarily applicable to only detection of well-recognized serotypes (de Filippis and McKee, 2012; Mostowy and Holt, 2018). With the advent of high-throughput sequencing technologies in research practice and in medical laboratories, there is a constant demand for robust bioinformatics tools utilizing genomic information to dissect serotype landscapes for characterization of pathogenic bacteria. *In silico* serotyping methods for several common pathogenic bacteria have been successfully developed (Mostowy and Holt, 2018). This approach offers alternative and validatory solutions to standard laboratory serotyping methods. Further innovation could prove helpful to bridging technological gaps between traditional serological serotyping and genomic typing in epidemiological investigation and diagnostic practice.

After reviewing literatures on *in silico* serotyping methods, we found that majority of the typing methods were based on two modes of strategies: identification by comparing the genome assembly or short reads with entire PS loci (PSgc) of reference serotype strains (i.e., full loci strategy) or identification by comparing the genome assembly or short reads with conserved genes in PSgc from reference serotype strains (i.e., conserved gene strategy). To date, there are nearly 100 known serotypes reported for *Streptococcus pneumoniae* based on differing antigenic properties of its capsule (Geno et al., 2015). By mapping whole-genome sequencing (WGS) reads to full-length reference CPSgc sequences for 92 serotypes with assistance from serogroup- or serotype-specific variation, Kapatai et al. (2016) developed an automated WGS-based serotyping bioinformatics tool, PneumoCaT (Pneumococcal Capsule Typing), which could predict serotype in 99% of the worldwide typeable isolates, with which prediction concordance with serologically derived serotypes reportedly increased to 99.3%. Recently, by using a database adapted from PneumoCaT and a k-mer-based method, Epping et al. (2018) developed a typing method of high computational performance, SeroBA, which reached up to 98% concordance with respect to traditional serotyping methods. Additionally, by using the whole O locus (namely, *rfb* gene cluster) and H antigen determination genes *fliC* and *fljB* as references databases, Zhang et al. developed SeqSero for *in silico Salmonella* serotyping based on high-throughput genome sequencing data and could theoretically identify 2,389 of the 2,577 serotypes described in the White–Kauffmann–Le Minor scheme (Zhang et al., 2015). Meanwhile, by adopting the conserved gene strategy, another *in silico* serotyping tool was developed for *Salmonella* comprising 246 serovars, SISTR (the *Salmonella In Silico* Typing Resource), which primarily targets O locus conserved genes including *wzx* and *wzy*, and H antigen determinant genes including *fliC* and *fljB*. Specially, SISTR *in silico* serotyping could incorporate cgMLST typing adjustment (Yoshida et al., 2016). For another well-known gram-negative pathogen *Pseudomonas aeruginosa*, *in silico* serotyping program PAst was developed, which covers 13 serogroups by alignment to the full-length reference sequences of O-specific antigen gene cluster (Thrane et al., 2016).

Several *in silico* serotyping tools were developed independently for *Escherichia coli* using similar a typing marker: conserved genes of O loci and the flagellin genes for H antigen. SerotypeFinder is a BLASTn-based prediction tool targeting *wzx*, *wzy*, *wzt*, and *wzm* for 185 O types, *fliC*, *flkA*, *flmA*, *flnA*, and *fllA* for all 53 H types of *E. coli* (Joensen et al., 2015); and another tool EBE incorporated O and H antigen prediction for *Shigella* in similar ways (Zhou and Fallows, 2021). Based on the reference genes collected in SerotypeFinder, Ingle et al., 2016 further curated an EcOH database that includes sequences of alleles of wzm and wzt, or wzx and wzy, covering 180 established O types (of a possible 182) and sequences for all 53 known H types, allowing for the detection of both *fliC* and non-*fliC* flagellin (*flnA*, *fmlA*, *flkA*, and *fllA*) genes. EcOH database was incorporated in SRST2 for *in silico* serotyping (Ingle et al., 2016). After characterizing the genetic diversity of K locus, Wyres et al. (2016) developed Kaptive using full-length K-loci as references for determining 134 K types of *Klebsiella*, and they then incorporated *in silico* O-locus typing using conserved *wzm* and *wzt* genes covering 11 O types (Wick et al., 2018).

The abovementioned typing methods have been widely used in subsequent genomic epidemiological studies on corresponding pathogens, as evidenced by high citation rates. The curated reference databases, nomenclature covering novel types, and typing methods presented in such *in silico* tool development studies are expected be progressively important as essential analytical resources for genomic surveillance and epidemiological investigations (Wyres et al., 2016). It is also anticipated that more sophisticated forms of *in silico* serotyping will be developed for an expanding scope of bacterial species, as the use of WGS becomes more common in clinical and laboratory-based studies.

PS loci are subject to environmental selection, and their mutations proceed faster than other genetic regions (Wyres et al., 2016; Rendueles et al., 2018; Holt et al., 2020). Many typing methods are designated with a match threshold of around 90% empirically, which is an identification threshold for biological species. The accuracy of the abovementioned typing methods is generally considered restricted by genetic variation of targeting loci or allele (as in full loci strategy). For example, if the genetic structure and gene content of PS loci of certain strain are conserved, and if its nucleotide sequences diverge largely to the extent of having below 90% identity to reference full-length loci (Holt et al., 2020), then it could become mis-typed. This is also true for the conserved gene strategy. Recombination variants between loci of different serogroups (Holt et al., 2020) will also get mis-typed using conserved gene strategy. New typing strategies could afford the development of more efficient typing methods. New genes may occur in PS loci of variant strains and may develop novel functions in PS biosynthesis and might further play roles in the emergence of novel populations, or even new serogroups. Thus, unique genes in PS loci of certain serotypes/serogroups are anticipated to be potentially useful *in silico* type markers (i.e., unique gene strategy), which could be less restricted as a result of a relaxed identity threshold and high specificity for uniqueness distribution. Thus far, the use of unique genes of a certain serogroup as assistant markers has only been reported for *Salmonella* (Zhang et al., 2015) and *S. pneumoniae* (Kapatai et al., 2016), whereas the use of unique genes as identification markers in serotyping remains unreported.

*Vibrio parahaemolyticus* has emerged as a globally important food-borne enteropathogen pathologically linked to causing acute gastroenteritis, wound infections, and septic shock (Ghenem et al., 2017). Like other pathogens, the antigenic properties of LPS (O antigen) and CPS (K antigen) provide a diagnostic basis for serotyping *V. parahaemolyticus*. Typically, serotyping assays rely on the use of commercially available antisera to identify and discriminate *V. parahaemolyticus* strains. Currently, 13 O group and 71 K types can be identified using commercial antisera (Oliver and Jones, 2015). However, this approach is inherently restricted by issues such as high costs, complicated procedures, cross-immunoreactivity, and subjective interpretation (Twedt et al., 1972). In a previous work by Chen and colleagues, the genetic structure of the LPS biosynthesis genetic cluster determining *V. parahaemolyticus* O serogroup was identified (Chen et al., 2012). In the meantime, the genetic structure of the CPS biosynthesis loci became gradually elucidated over the past two decades (Guvener and McCarter, 2003; Okura et al., 2008; Chen et al., 2010; Pang et al., 2019). Recently, our group identified and characterized the genetic structure of whole CPS loci by expanding our analytical scope into a new 3′ border gene, *glpX*, which is conserved among all K serogroups (Bian et al., 2020). Collectively, these efforts on clarifying *V. parahaemolyticus* PSgcs have made *in silico* serotyping with WGS data possible. Previously, along the development of the PCR-based molecular serotyping method, Pang et al. developed a program for *in silico* classification of 55 K serogroups based on a conserved gene strategy targeting the *wzy* (52 K serogroups) and *wzx* (K22, K52, and K60) genes, with a threshold of 98% and a minimum length of 95% (Pang et al., 2019). However, no accuracy details for each serogroup were tested or reported.

Incorporating novel genes from other species through recombination may play instrumental roles in the evolution and divergence of PS loci in *V. parahaemolyticus*, reflecting emergence of novel serogroup populations (Guo et al., 2017; Bian et al., 2020). Indeed, unique serogroup-specific gens can also be found in most serogroups (Chen et al., 2012; Bian et al., 2020). In order to prove the feasibility and test the robustness of unique genes in *in silico* typing, in the current study, we made comparisons between LPSgc and CPSgc from 43 K and all 12 O serogroups, identified serogroup-specific genes, and developed an *in silico* algorithm VPsero using these genes as markers. VPsero predicted serogroup and serotypes from assembled draft genomes and displayed high specificity and sensitivity in prediction toward a testing set of *V. parahaemolyticus* strains. VPsero could also report potential novel serogroups or serotypes, which is important for downstream validation and allows for much deeper investigation. VPsero is anticipated to be of high utility for dissecting the genetic diversity of LPS and CPS.

## Materials and Methods

### Genomic Data of *Vibrio parahaemolyticus*, and LPSgc/CPSgc Identification

Genome data of 443 *V. parahaemolyticus* strains were retrieved from our previous study (Bian et al., 2020), and other 1,103 *V. parahaemolyticus* strains were downloaded from GenBank, based on works by Yang et al. (2019). Draft genomes were subject to analysis for identification of CPS and LPS gene cluster sequences, as described below. Briefly, coding sequences (CDS) of each strain were initially predicted using prokka 1.13 (Seemann, 2014) against Swiss-Prot database in UniProt. For CPS gene cluster, gene *gmhD* and gene *rjg* were chosen as the 5′ and 3′ border genes, as described previously (Bian et al., 2020). For each strain, if a certain contig contained both the 5′ and 3′ border genes, then a putative whole capsule gene cluster would be extracted from this contig. For LPS gene cluster, similar methods were adopted using gene *VP0190* as the 5′ border gene and gene *gmhD* as the 3′ border gene (Okura et al., 2008). Border genes were queried using Blast (Madden, 2013) with the following parameters: e-value lower than 1e-5, identity greater than 60%, and coverage greater than 60%.

### Identification and Annotation of Homologous Gene in Representative Gene Clusters for Polysaccharide

Functional annotation of open reading frames (ORFs) of representative LPS and CPS gene clusters was conducted, as described in our previous study (Bian et al., 2020). Briefly, after LPSgc and CPSgc were determined as in *Genomic Data of Vibrio parahaemolyticus, and Gene Cluster for Lipopolysaccharide/Gene Cluster for Capsular Polysaccharide Identification*, all ORFs of these representative PSgcs were clustered by OrthoFinder with default parameters and assigned to orthogroups (Emms and Kelly, 2019). Each orthogroup was designated as a gene group and was uniformly designated as follows: all ORFs were annotated using prokka, Swiss-Prot, or our previous identified orthogroups (Bian et al., 2020). Genes with the largest proportion were chosen as the name of a particular orthogroup. For orthogroups that could not be annotated or named using the aforementioned method, the orthogroup ID generated by OrthoFinder was used as its name.

### VPsero Workflow

**FIGURE 1:**
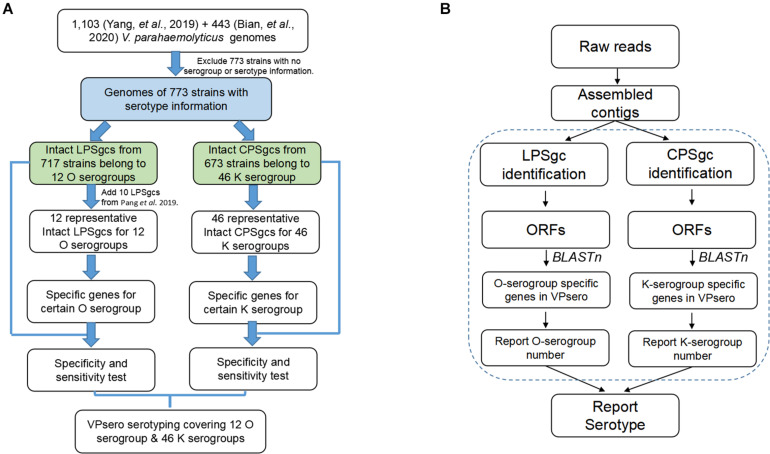
Flowchart outlining **(A)** development process of VPsero and **(B)** algorithm of VPsero.

The work-flow is displayed in Figure 1B. After the presence of full-length LPS or CPS gene clusters was determined as in *Identification and Annotation of Homologous Gene in Representative Gene Clusters for Polysaccharide*, all ORFs of LPS and CPS gene clusters were subjected to BLASTn analysis against the reference marker genes of O and K serogroups, respectively, with an identity threshold of 80% and a coverage threshold of 80%. Finally, the O and K combinations were recognized as serotype Ox:Ky, while non-typeable serogroups using VPsero were denoted as Ont or Knt (nt means “not typeable”). For the strains whose PSgc cannot be identified because of absence or sequencing quality, they were reported as One/Kne (“ne” means “can*n*ot be *e*xtracted”).

### Specificity and Sensitivity of *in silico* Typing Assessment

Serotyped O and K strains were used for specificity and sensitivity assessment of O and K serogroup *in silico* typing, respectively.

For K serogroups,

Sensitivity =VPsero_typed K_*i*_/(VPsero_typed K_*i*_ + VPsero_ untyped K_*i*_)

where K_*i*_ indicates certain K serogroup. Sensitivity was assessed using certain serotyped K_*i*_ strains determined by immunological serum, where VPsero_typed K_*i*_ and VPsero_untyped K_*i*_ are the numbers of strains capable of being typed and untyped by Vpsero, respectively.

Specificity =Serotyped no-K_*i*_/(Serotyped no-K_*i*_ + VPsero_ typed K_*i*_)

Specificity was assessed using no-K_*i*_ serogroup strains (Serotyped no-K_*i*_), which do not belong to K_*i*_ serogroups, as determined by immunological serum; VPsero_typed K_*i*_ is the number of strains being typed as K_*i*_ serogroup using VPsero. O serogroups and serotypes were assessed as in abovementioned similar ways.

## Results

### Potential Marker Genes for O and K Serogroups

**FIGURE 2:**
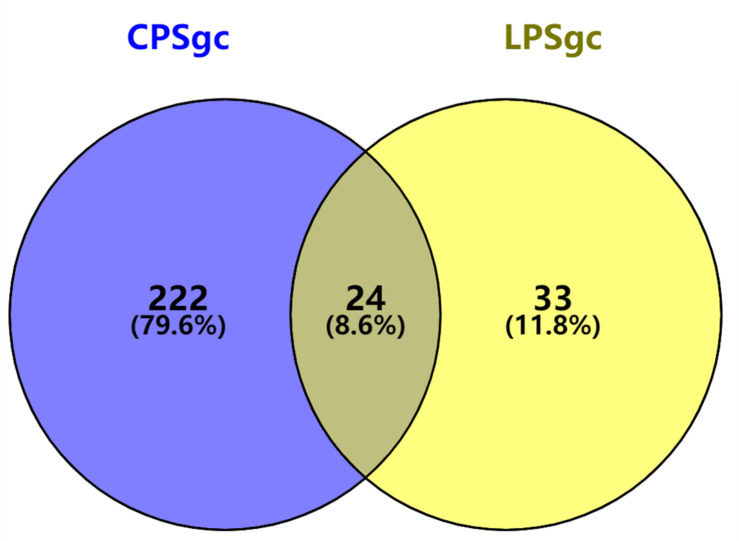
Shared and unique gene orthogroups identified in gene clusters for lipopolysaccharide (LPSgc) and gene clusters for capsular polysaccharide (CPSgc).

Seven hundred seventeen strains with intact LPSgcs covering 12 O serogroups (Supplementary Table 1) and 673 strains with intact CPSgcs covering 46 K serogroups (Supplementary Table 2) were identified from 1,546 *V. parahaemolyticus* genomes (Yang et al., 2019; Bian et al., 2020). These were selected for subsequent algorithm development and tests (Figure 1A). Twelve O serogroups’ representative LPSgcs and 46 K serogroups’ representative CPSgcs (Supplementary Tables 4, 5) were selected by comparing gene contents of PSgcs annotated using prokka 1.13 (Seemann, 2014) in heatmaps (data not shown). For identification of unique genes for each O and K serogroup, we re-annotated ORFs of representative CPSgcs by homology using OrthoFinder. A total of 260 gene orthogroups could be annotated from these representative gene clusters (Supplementary Table 3). Nomenclature or numbering of these orthogroups is compatible with our previous study (Bian et al., 2020). Numbering of gene orthogroups was first recognized in Bian et al. (2020) and was kept as is in this study, while numbering of novel gene orthogroups was assigned using OrthoFinder. Among these gene orthogroups, only 33 appear in LPSgcs. Majority of these gene groups (203) are from CPSgcs, and 24 were shared by LPSgc and CPSgc (Figure 2 and Supplementary Table 3).

Subsequently, potential marker genes that are only unique for certain O or K serogroup were identified. For majority of the serogroups, at least one potential marker gene was identified. Nineteen potential marker genes were identified for 10 O serogroups (Supplementary Table 4), while 104 potential marker genes were identified for 43 K serogroups (Supplementary Table 5). More than one potential marker gene was identified in 31 K and four O serogroups. For the five serogroups O12, O7, K55, K23K, and K37, in which no unique genes could be identified, we adopted another strategy: two genes in combination that are unique for one serogroup were selected as combinatorial marker genes (Supplementary Tables 4, 5).

### *In silico* Serogroup Typing Assessment

**FIGURE 3:**
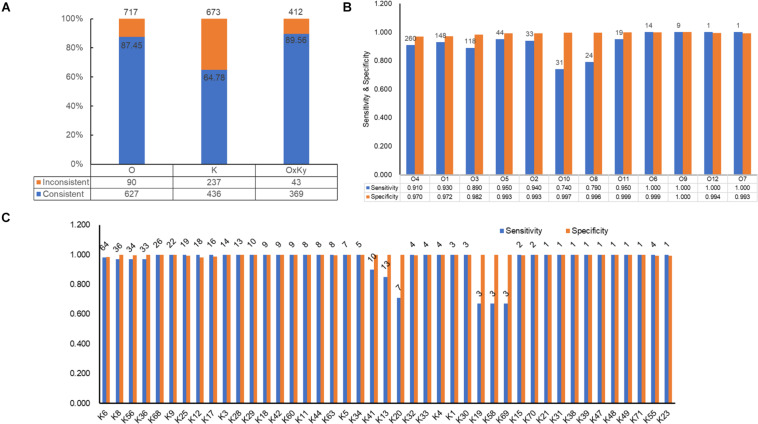
Identification performance of VPsero. **(A)** Consistency between serological determination and VPsero *in silico* identification by O and K serogroups and serotypes. **(B)** Sensitivity and specificity of VPsero in identification of 12 O serogroups. **(C)** Sensitivity and specificity of VPsero in identification of 43 K serogroups. Total numbers of strains tested in **(B,C)** are as indicated above for each serogroup.

For selecting an optimal marker gene, the above potential marker genes were subjected to *in silico* serogroup typing tests. O and K serogroup markers were tested in 717 strains and 673 strains, respectively (Figure 3A and Supplementary Tables 1, 2). All potential marker genes of 12 O serogroups passed test with high specificity, but only OG266 for O10 (Figure 3B and Supplementary Table 4). Similarly, for K serogroups, most potential marker genes were found qualified; but two genes for K17, one gene for K63, two genes for K5, and all potential marker genes for K37, K10, and K53 did not pass the test (Figure 3C and Supplementary Table 5). Among these qualified markers, for each serogroup, genes permitting the highest sensitivity were selected for serogroup prediction by VPsero (Tables 1, 2). When sensitivity was the same for two or more marker genes, the one with higher specificity was selected. Sensitivity toward these chosen typing markers varied from 0.667 to 1.000 and showed a negative correlation with strain numbers, which suggests that inaccuracy may arise from insufficient sampling. For robustness and reference, we added a prefix “p” (meaning putative) for reporting these insufficient sampled serogroups (Tables 1, 2). The average sensitivity of VPsero for serogroup prediction is 0.910 for O and 0.961 for K serogroups. The average specificity is 0.990 and 0.998 for O and K serogroups, respectively (Tables 1, 2 and Figures 3B,C). In summary, our results show that VPsero is well applicable in the prediction of 12 O and 43 K serogroups and can perform with high accuracy.

**TABLE 1:** O serogroup typing genes by VPsero and corresponding sensitivity and specificity.

No.	O serogroup	Strain number	Typing gene	Predication model	Sensitivity	Specificity	Report serogroup
1	O4	260	*Rv3806c*	One gene	0.910	0.970	O4
2	O1	148	*phaJ*	One gene	0.930	0.972	O1
3	O3	118	*yafV*	One gene	0.890	0.982	O3
4	O5	44	*arnA*	One gene	0.950	0.993	O5
5	O2	33	*yejM*	One gene	0.940	0.993	O2
6	O10	31	OG265	One gene	0.740	0.997	O10
7	O8	24	*pbgP3*	One gene	0.790	0.996	O8
8	O11	19	OG267	One gene	0.950	0.999	O11
9	O6	14	OG280	One gene	1.000	0.999	O6
10	O9	9	OG283	One gene	1.000	1.000	O9
11	O12	1	OG247&*yvfD*	Two genes	1.000	0.994	pO12
12	O7	1	OG247&OG219	Two genes	1.000	0.993	pO7

**TABLE 2:** K serogroup typing genes by VPsero and corresponding sensitivity and specificity.

No.	K serogroup	Strain number	Typing gene	Predication model	Sensitivity	Specificity	Report serogroup
1	K6	64	VP0226	One gene	0.980	0.983	K6
2	K8	36	OG161	One gene	0.970	0.998	K8
3	K56	34	OG203	One gene	0.970	0.997	K56
4	K36	33	OG128	One gene	0.970	1.000	K36
5	K68	26	OG154	One gene	1.000	1.000	K68
6	K9	22	OG146	One gene	1.000	0.998	K9
7	K25	19	OG142	One gene	1.000	0.992	K25
8	K12	18	OG158	One gene	1.000	0.982	K12
9	K17	16	OG209	One gene	1.000	0.987	K17
10	K3	14	OG193	One gene	1.000	1.000	K3
11	K28	13	OG216	One gene	1.000	1.000	K28
12	K29	10	OG195	One gene	1.000	1.000	K29
13	K18	9	OG174	One gene	1.000	0.998	K18
14	K42	9	OG205	One gene	1.000	0.998	K42
15	K60	9	*pseG*	One gene	1.000	1.000	K60
16	K11	8	OG131	One gene	1.000	1.000	K11
17	K44	8	OG138	One gene	1.000	1.000	K44
18	K63	8	OG198	One gene	1.000	0.997	K63
19	K5	7	OG169	One gene	1.000	0.998	K5
20	K34	5	OG217	One gene	1.000	0.998	K34
21	K41	10	OG189	One gene	0.900	0.998	K41
22	K13	13	OG134	One gene	0.850	1.000	K13
23	K20	7	OG252	One gene	0.710	1.000	K20
24	K32	4	*fdtA*	One gene	1.000	0.997	*pK32*
25	K33	4	OG183	One gene	1.000	1.000	*pK33*
26	K4	4	OG177	One gene	1.000	1.000	*pK4*
27	K1	3	OG180	One gene	1.000	1.000	*pK1*
28	K30	3	OG164	One gene	1.000	0.998	*pK30*
29	K19	3	OG166	One gene	0.670	1.000	*pK19*
30	K58	3	OG260	One gene	0.670	1.000	*pK58*
31	K69	3	OG201	One gene	0.670	1.000	*pK69*
32	K15	2	OG251	One gene	1.000	0.997	*pK15*
33	K70	2	*yveQ*	One gene	1.000	0.998	*pK70*
34	K21	1	OG212	One gene	1.000	1.000	*pK21*
35	K31	1	*murB*	One gene	1.000	1.000	*pK31*
36	K38	1	OG181	One gene	1.000	0.998	*pK38*
37	K39	1	OG253	One gene	1.000	1.000	*pK39*
38	K47	1	OG256	One gene	1.000	1.000	*pK47*
39	K48	1	OG186	One gene	1.000	1.000	*pK48*
40	K49	1	OG191	One gene	1.000	1.000	*pK49*
41	K71	1	OG197	One gene	1.000	1.000	*pK71*
42	K55	4	OG071&OG58	Two genes	1.000	0.994	*pK55*
43	K23	1	OG082&OG080	Two genes	1.000	0.992	*pK23*
44	K37	3	−	Two genes	−	−	−
45	K10	2	−	One gene	−	−	−
46	K53	2	−	One gene	−	−	−

### *In silico* Serotyping Assessment

*In silico* serotyping assessment was done in 412 strains with both intact LPSgc and CPSgc and with concrete serotype information, encompassing 62 serologically tested serotypes (Table 3, Figure 3A and Supplementary Table 6). Specificity for all serotypes reached above 0.99. Sensitivity for 22 serotypes with more than five strains tested reached 1.00 but is lower for O4:K68 (0.8077), O4:K13 (0.9167), O4:K63 (0.3750), and O8:K41 (0.8000). For the 40 serotypes with less than five strains, sensitivity for 18 serotypes reached 1.00, and 21 serotypes could not be typed correctly, which may be attributed to limited strain numbers that overshadow statistical significance and thus should be assessed additionally in the future when more genomic data of serotypes become available. Among these inconsistent typing, nine serotypes were caused by O4 inconsistent typing, four serotypes by O3, two serotypes by O8, and three serotypes by O1. Eleven serotypes were caused by various forms of inconsistent K typing (Table 3 and Supplementary Table 6). The inconsistency between serological typing and *in silico* typing is mainly due to serologically mis-serotyping (see analysis below). Collectively, VPsero performs robustly in *in silico* typing serotypes combined with 12 O and 43 K serogroups.

**TABLE 3:** Sensitivity and specificity for 21 serotypes identification using VPsero.

No.	Serotype	Strain numbers*	Sensitivity	Specificity	Predicting Serotype distribution ^#^
1	*O1:K36*	32	1.0000	1.0000	O1:K36-1.0
2	O1:K56	27	1.0000	0.9948	O1:K56-1.0
3	O1:K25	17	1.0000	0.9949	O1:K25-1.0
4	O1:K20	5	1.0000	1.0000	O1:K20-1.0
5	O2:K3	12	1.0000	0.9950	O2:K3-1.0
6	O2:K28	11	1.0000	0.9975	O2:K28-1.0
7	O3:K6	45	1.0000	0.9973	O3:K6-1.0
8	O3:K29	10	1.0000	1.0000	O3:K29-1.0
9	O3:K5	7	1.0000	1.0000	O3:K5-1.0
10	O4:K8	32	1.0000	0.9921	O4:K8-1.0
11	O4:K9	22	1.0000	1.0000	O4:K9-1.0
12	O4:K12	17	1.0000	0.9975	O4:K12-1.0
13	O4:K42	9	1.0000	1.0000	O4:K42-1.0
14	O4:K11	7	1.0000	1.0000	O4:K11-1.0
15	O5:K17	15	1.0000	0.9975	O5:K17-1.0
16	O6:K18	9	1.0000	1.0000	O6:K18-1.0
17	O9:K44	8	1.0000	1.0000	O9:K44-1.0
18	*O10:K60*	8	1.0000	0.9975	O10:K60-1.0
19	**O4**:K68	26	0.8077	1.0000	O4:K68-0.808,Ont:K68-0.192
20	O4:**K13**	12	0.9167	1.0000	O4:K13-0.917,O4:K12-0.083
21	**O4**:K63	8	0.3750	1.0000	Ont:K63-0.625,O4:K63-0.375
22	**O8:K41**	10	0.8000	1.0000	O8:K41-0.8,O1:K41-0.1,O5:K17-0.1
Average			0.9500	0.9984	

*22 serotypes with more than 5 strains that could be test are displayed. Serogroups occurred mis-predication are in bold font in the **Serotype** column and **Predicting Serotype distribution** column respectively. ^∗^ Number of Strains with both intact LPSgc and CPSgc. # Frequency of predicting serotype are listed.*

### Sources of Inconsistency Between Serological Typing and *in silico* Typing

Furthermore, to determine the factors leading to inconsistency in *in silico* typing with respect to wet-lab typing (Figure 3A), we listed all inconsistent typing pairs by serogroups (Supplementary Tables 7, 8) and carried out comparative analysis on their gene contents of PSgcs among inconsistently typed strains and representative strains. We found that inconsistencies arise mainly due to serological mis-serotyping, as judged by the similarity between PSgcs gene contents and representative references (Supplementary Data 1, Supplementary Data 2). For convenience of analysis, the serologically mis-serotyped ones were classified into two classes, namely, those that could be typed by VPsero and those that could not be typed by VPsero (Ont/Knt). Sixty in 90 inconsistent O-serogroup strains and 84 in 237 inconsistent K-serogroup strains was correctly typed by VPsero, which is also supported by similarity in gene contents and lengths of PSgcs (Sheets 1 of Supplementary Data 1 and Supplementary Data 2). O4, O1, O3, O8, and O10 account for most of the mis-serotyped O serogroup strains, while the serogroup-unresolvable K strains (51) and KUT strains (28) account for most of the mis-serotyped K serogroup strains (Supplementary Tables 7, 8; sheet 1, 2 of Supplementary Data 1 and Supplementary Data 2). Upon confirmation of serogroups of the strains through repeating serological tests, it would be expected that the sensitivity and specificity of corresponding serogroups and thus serotypes could be improved methodologically.

Thirty in 90 inconsistent O-serogroup strains and 153 in 237 inconsistent K-serogroup strains were *in silico* typed as Ont and Knt, respectively. Similarly, the mis-typed O4, O1, O8, and O10 account for most of the non-typeable O strains by VPsero; while KUT (94) and the serogroup-unresolvable K strains (49) account for majority of the non-typeable K strains (Supplementary Tables 7, 8; sheets 1 of Supplementary Data 1 and Supplementary Data 2). We proposed that the serogroups uncovered by VPsero and the emergence of population differentiation of certain serogroups or novel serogroup populations (Guo et al., 2017) could be another possible reason for these serogroup inconsistency. Four uncharacterized O-serogroup populations, O_pop1–4, were recognized by similarity of genetic contents and lengths of LPSgcs. Among them, O_pop3,4 are potential variants of O4 serogroups. Likewise, 24 uncharacterized K-serogroup populations, K_pop1–24, were recognized. Most of them could be serogroups uncovered by VPsero or the subpopulations of certain K-serogroup or novel K-serogroup populations, except for K_pop4 and K_pop14, which are possible variants of K5 and K53, respectively. Thus, VPsero could be a useful tool for identifying potential serogroup variants and even novel serogroups, by indicating PSgc genetic content/structural variations. This makes the algorithm of much value to clinical and basic research investigation.

The third reason for the inconsistency and lower sensitivity or specificity concerns a shortage of strains with correct serogroup information to cover diversity and to reveal the right representative PSgcs in a true population of some serogroup, especially the K serogroups such as K13, K41, K58, K20, K37, K10, and K19 (Table 2 and Supplementary Data 2). This issue warrants future investigation when more genomic data of the serogroups become available.

## Discussion

In this study, we presented VPsero as a novel tool for rapid determination of serotypes of *V. parahaemolyticus* using serogroup-specific genes, based on whole genome or polysaccharide biosynthesis locus sequencing data. We also proved that unique-gene strategy is viable for typing, which could lead to less constraints with relaxed identity thresholds as well as higher specificity. In VPsero, a key serogroup-specific marker is conserved in terms of sequence and function in the corresponding serogroup, wherein a general default threshold of 80% will prove sufficient for *in silico* serotyping. Notably, *in silico* serotyping is anticipated to be useful for guiding or updating serological typing to have a broader coverage on prevailing pathogens. It should also be helpful for investigation on evolutionary synergy between the antigen diversity of LPS/CPS and their genetic background of corresponding pathogenic bacteria. *In silico* serotyping utilizing/incorporating the unique-gene strategy has the clear merits of being a high-sensitivity approach to systematic investigation.

Polysaccharides are major virulence factors and thus an important vaccine target in various bacterial pathogens (Szymanski et al., 2015; Mistou et al., 2016). They are always under selective pressure imposed by, the host or the environment. Genetic variations of PSgc thus occur to give rise to polysaccharide structural diversification and escape of the pathogens from evolutionary stress (Rendueles et al., 2018; Holt et al., 2020). Antigenic diversity and epidemiology of polysaccharides, and thus genetic diversity of the biosynthetic determinant PSgcs, are of fundamental importance to bacterial pathogenesis and public health (van Tonder et al., 2016; Mostowy and Holt, 2018). The emergence of new CPSgcs in *V. parahaemolyticus* was found through gene recombination or duplication (Guo et al., 2017; Bian et al., 2020). Serological non-typeable strains irrespective the serological mistyping are frequently found during routine laboratory detection (Jones et al., 2012; Gavilan et al., 2013; Siddique et al., 2021) and display a higher level of diversity than expected (Shengzhe, 2020). The emergence mechanisms, genetic diversity, and epidemiology of KUT and OUT are not well recognized until recently. In this study, we differentiated the serological non-typeables (OUT, KUT) and the those that could not be typed using VPsero, labeled as Ont and Knt. From our analysis, majority of the OUTs and part of KUT, 22 of 25 OUT strains and 26 of 120 KUT, might be serological mis-typed; and the other OUT/KUT are also not typeable using VPsero (Supplementary Tables 7, 8; Supplementary Data 1, Supplementary Data 2). On the other hand, part of these potential mis-type strains might exactly variant of known serogroup that could not be typed serologically but not mis-typed. Based on our results, in the contexts of *V. parahaemolyticus* surveillance and accompanied genomic characterization, VPsero could be of high utility in serotyping, on the ground of being a less subjective typing approach and capable of recognizing new epidemic populations with high accuracy in the genomic epidemiology era (Balloux et al., 2018). In the future, for better compatibility and accuracy afforded by refined methods, it is anticipated that novel populations can be defined by integrating information on serotyping and genetic structural contents, possibly with the assistance of phylogenetic relationship analysis.

VPsero covers major prevalent O and K serogroups, but not all serogroups (Oliver and Jones, 2015). Additionally, marker genes of a number of O and K serogroups (namely, 2 O and 29 K serogroups) were tested with a relatively small number of strains (Tables 1, 2). Should additional data including more serogroups become available, larger sets of strains could be tested to improve the robustness of the algorithm, and more serogroups can be implanted. Additionally, during development, we found that the quality of assembled genomes was considerably influenced by sequencing depths, which are key to successful typing (Wyres et al., 2020). We recommend that the sequencing depths be above 100 empirically based our own data. Another potential limitation of the current version of VPsero lies in that only assembled genomes or contigs but not short reads are potentially applicable for serotyping, which warrants efforts on future improvements.

## Data Availability Statement

The original contributions presented in the study are included in the article/Supplementary Material, further inquiries can be directed to the corresponding author/s.

## Author Contributions

LL, SB, YZ, and QH were involved in the conceptualization. SB, LL, and QZ were involved in the data curation and the formal analysis. LL and YZ were involved in the funding acquisition. LL and YJ were involved in the visualization and writing–original draft. LL, YJ, and N-KW were involved in writing–review & editing. All authors contributed to the article and approved the submitted version.

## Conflict of Interest

The authors declare that the research was conducted in the absence of any commercial or financial relationships that could be construed as a potential conflict of interest.

## Publisher’s Note

All claims expressed in this article are solely those of the authors and do not necessarily represent those of their affiliated organizations, or those of the publisher, the editors and the reviewers. Any product that may be evaluated in this article, or claim that may be made by its manufacturer, is not guaranteed or endorsed by the publisher.

## Supplementary Material

Supplementary Table 1 Strains used for identification of O serogroup marker genes and tests on the algorithm in this study. Note:^∗^ GenBank (with prefix “GCA”) or CNGB (with prefix “CNA”) accession numbers for assembled genomes or sequences that harbor LPS gene clusters.

Supplementary Table 2 Strains used in identification of K serogroup marker genes and tests on the algorithm in this study. Note: ^∗^ GenBank (with prefix “GCA”) or CNGB (with prefix “CNA”) accession number for assembled genomes or sequences that harbor LPS gene clusters.

Supplementary Table 3 Gene orthogroups identified in this stud.

Supplementary Table 4 Potential O serogroup marker genes.

Supplementary Table 5 Potential K serogroup marker genes.

Supplementary Table 6 Sensitivity and specificity in serotype identification by VPsero. Note: 40 serotypes with more than 5 strains which were testable are displayed. Mis-predication could arise in serogroups. The mis-predicted serotypes are diaplyed in red font in the Serotype column and Predicted serotype distribution column, respectively. ^∗^ Numbers of strains with both intact LPSgc and CPSgc. # Frequencies of predicted serotype are listed.

Supplementary Table 7 Constitution of VPsero-identified O serogroups that were inconsistent with serological determination.

Supplementary Table 8 Constitution of VPsero-identified K serogroups that were inconsistent with serological determination.

Supplementary Data 1 Cluster of LPSgc gene contents for strains that may contain incorrect serological O serogroups. Note: Strains listed in this dataset were subjected to identification of O serogroups by Vpsero, which turned out to be inconsistent with serological determination. Coding sequences (CDS) of LPSgc for each strain were annotated using prokka 1.13 against Swiss-Prot database in UniProt (as described in Materials and Methods). Each column indicates one gene which corresponds to one Uniprot ID. Copy numbers of these genes are indicated in each cell. Coding sequences that cannot be annotated against the Swiss-Prot database are not displayed in this dataset. Representative LPSgc strains with red and bold font are also listed for reference. Strains were sorted by length of LPSgc, and presentation of the results was manually adjusted according the genetic contents similarity. Sheet1 (1.known_O_serogroups) displays the ones that can be identified as certain K serogroups covered by Vpsero. Sheet2 (2.Ont) displays the ones that cannot be identified by VPsero and are thus labeled as Ont. Ont strains, at least 2, with similar contents are recognized as an uncharacterized O-serogroup pop8ulation for clarity, and 4 populations (O_pop1-4) are as indicated in Sheet2.

Supplementary Data 2 Cluster of CPSgc gene contents for strains that may contain incorrect serological K serogroups. Note: Strains listed in this dataset are identified as K serogroups by using Vpsero, which were inconsistent with serological determination. Gene annotated as described in Supplementary Data 1 | Strains are sorted by length of CPSgc and presentation of results was manually adjusted according genetic contents similarity. Representative CPSgc strains with red and bold font are also listed for reference. Sheet1 displays the ones that can be identified as certain K serogroup scovered by Vpsero. Sheet2 displays the ones that cannot be identified by VPsero and are thus labeled as Ont. As in the case of Ont strains, 24 uncharaterized K-serogroup populations (K_pop1-24) are indicated in Sheet2.
