# Supplementary material for: VPsero: Rapid Serotyping of Vibrio parahaemolyticus Using Serogroup-Specific Genes Based on Whole-Genome Sequencing Data
Source: Front Microbiol. 2021 Sep 2;12:620224. doi: 10.3389/fmicb.2021.620224 (PMC8443796; doi:10.3389/fmicb.2021.620224)
Supplement: Supplementary Table 1 — Strains used for identification of O serogroup marker genes and tests on the algorithm in this study. Note:∗ GenBank (with prefix “GCA”) or CNGB (with prefix “CNA”) accession numbers for assembled genomes or sequences that harbor LPS gene clusters. [file Presentation_1.zip › Supplementary_tables/Table S6.docx]

**Supplementary Table S6.**

|  | **Serotype** | **Strain numbers*** | **Sensitivity** | **Specificity** | **Predicting Serotype distribution** |
| --- | --- | --- | --- | --- | --- |
| 1 | O1:K1 | 3 | 1.0000 | 1.0000 | O1:K1-1.0 |
| 2 | *O1:K23* | 1 | 1.0000 | 1.0000 | O1:K23-1.0 |
| 3 | *O1:K33* | 2 | 1.0000 | 0.9976 | O1:K33-1.0 |
| 4 | O1:K38 | 1 | 1.0000 | 1.0000 | O1:K38-1.0 |
| 5 | O1:K58 | 2 | 1.0000 | 1.0000 | O1:K58-1.0 |
| 6 | O1:K69 | 1 | 1.0000 | 1.0000 | O1:K69-1.0 |
| 7 | O10:K71 | 1 | 1.0000 | 1.0000 | O10:K71-1.0 |
| 8 | O11:K19 | 2 | 1.0000 | 1.0000 | O11:K19-1.0 |
| 9 | O3:K31 | 1 | 1.0000 | 1.0000 | O3:K31-1.0 |
| 10 | *O3:K39* | 1 | 1.0000 | 1.0000 | O3:K39-1.0 |
| 11 | O3:K48 | 1 | 1.0000 | 1.0000 | O3:K48-1.0 |
| 12 | O4:K4 | 4 | 1.0000 | 1.0000 | O4:K4-1.0 |
| 13 | O4:K49 | 1 | 1.0000 | 1.0000 | O4:K49-1.0 |
| 14 | O4:K55 | 3 | 1.0000 | 0.9976 | O4:K55-1.0 |
| 15 | O5:K30 | 3 | 1.0000 | 1.0000 | O5:K30-1.0 |
| 16 | O5:K47 | 1 | 1.0000 | 1.0000 | O5:K47-1.0 |
| 17 | O8:K21 | 1 | 1.0000 | 1.0000 | O8:K21-1.0 |
| 18 | O8:K70 | 2 | 1.0000 | 1.0000 | O8:K70-1.0 |
| 19 | **O4**:K34 | 4 | 0.5000 | 1.0000 | O4:K34-0.5, Ont:K34-0.5 |
| 20 | **O1**:K32 | 3 | 0.0000 | 1.0000 | Ont:K32-1.0 |
| 21 | **O1**:K55 | 1 | 0.0000 | 1.0000 | O4:K55-1.0 |
| 22 | **O1**:K8 | 1 | 0.0000 | 1.0000 | O4:K8-1.0 |
| 23 | **O10**:**K19** | 1 | 0.0000 | 1.0000 | O12:Knt-1.0 |
| 24 | O11:**K20** | 2 | 0.0000 | 1.0000 | O11:Knt-1.0 |
| 25 | O11**:K36** | 1 | 0.0000 | 1.0000 | O11:Knt-1.0 |
| 26 | **O3**:K3 | 2 | 0.0000 | 1.0000 | O2:K3-1.0 |
| 27 | **O3:K37** | 2 | 0.0000 | 1.0000 | O3:Knt-0.5,O4:Knt-0.5 |
| 28 | **O3**:K56 | 2 | 0.0000 | 1.0000 | O1:K56-0.5,O5:K56-0.5 |
| 29 | O3:**K58** | 1 | 0.0000 | 1.0000 | O3:K6-1.0 |
| 30 | **O3**:K8 | 1 | 0.0000 | 1.0000 | O4:K8-1.0 |
| 31 | O4:**K10** | 1 | 0.0000 | 1.0000 | O4:Knt-1.0 |
| 32 | **O4**:K25 | 2 | 0.0000 | 1.0000 | O1:K25-1.0 |
| 33 | **O4**:K28 | 1 | 0.0000 | 1.0000 | O2:K28-1.0 |
| 34 | **O4**:K33 | 1 | 0.0000 | 1.0000 | O1:K33-1.0 |
| 35 | **O4:K36** | 1 | 0.0000 | 1.0000 | O5:Knt-1.0 |
| 36 | O4:**K37** | 1 | 0.0000 | 1.0000 | O4:Knt-1.0 |
| 37 | O4:**K53** | 1 | 0.0000 | 1.0000 | O4:Knt-1.0 |
| 38 | **O4**:K56 | 1 | 0.0000 | 1.0000 | O1:K56-1.0 |
| 39 | **O4**:K60 | 1 | 0.0000 | 1.0000 | O10:K60-1.0 |
| 40 | **O8**:K8 | 1 | 0.0000 | 1.0000 | O4:K8-1.0 |

Note: 40 serotypes with more than 5 strains that could be test are displayed. Serogroups occurred mis-predication and their mis-predicted serotypes are in red font in the **Serotype** column and **Predicting Serotype distribution** column respectively. * Number of Strains with both intact LPSgc and CPSgc. # Frequency of predicting serotype are listed.
