# Supplementary material for: VPsero: Rapid Serotyping of Vibrio parahaemolyticus Using Serogroup-Specific Genes Based on Whole-Genome Sequencing Data
Source: Front Microbiol. 2021 Sep 2;12:620224. doi: 10.3389/fmicb.2021.620224 (PMC8443796; doi:10.3389/fmicb.2021.620224)
Supplement: Supplementary Table 1 — Strains used for identification of O serogroup marker genes and tests on the algorithm in this study. Note:∗ GenBank (with prefix “GCA”) or CNGB (with prefix “CNA”) accession numbers for assembled genomes or sequences that harbor LPS gene clusters. [file Presentation_1.zip › Supplementary_tables/Table S7.docx]

**Supplementary Table S7.**

| **Transition pair** | **Predict O serogroup** | **Strain number** | **Strain number by predict O serogroup** |
| --- | --- | --- | --- |
| O4-O1 | O1 | 5 | 15 |
| O3-O1 | O1 | 2 |  |
| O8-O1 | O1 | 1 |  |
| OUT-O1 | O1 | 7 |  |
| O3-O4 | O4 | 5 | 13 |
| O1-O4 | O4 | 2 |  |
| O8-O4 | O4 | 1 |  |
| OUT-O4 | O4 | 5 |  |
| O4-O3 | O3 | 1 | 11 |
| O1-O3 | O3 | 2 |  |
| O2-O3 | O3 | 1 |  |
| O5-O3 | O3 | 1 |  |
| O11-O3 | O3 | 1 |  |
| OUT-O3 | O3 | 5 |  |
| O4-O2 | O2 | 1 | 5 |
| O3-O2 | O2 | 2 |  |
| OUT-O2 | O2 | 2 |  |
| O10-O12 | O12 | 4 | 4 |
| O4-O5 | O5 | 1 | 4 |
| O3-O5 | O5 | 1 |  |
| O8-O5 | O5 | 1 |  |
| OUT-O5 | O5 | 1 |  |
| O3-O8 | O8 | 1 | 3 |
| OUT-O8 | O8 | 2 |  |
| O4-O10 | O10 | 1 | 2 |
| O3-O10 | O10 | 1 |  |
| O4-O11 | O11 | 1 | 1 |
| O10-O6 | O6 | 1 | 1 |
| O10-O7 | O7 | 1 | 1 |
| O4-Ont | Ont | 18 | 30 |
| O1-Ont | Ont | 5 |  |
| O10-Ont | Ont | 1 |  |
| O8-Ont | Ont | 2 |  |
| O2-Ont | Ont | 1 |  |
| OUT-Ont | Ont | 3 |  |
| Total |  | 90 | |
