# Supplementary material for: VPsero: Rapid Serotyping of Vibrio parahaemolyticus Using Serogroup-Specific Genes Based on Whole-Genome Sequencing Data
Source: Front Microbiol. 2021 Sep 2;12:620224. doi: 10.3389/fmicb.2021.620224 (PMC8443796; doi:10.3389/fmicb.2021.620224)
Supplement: Supplementary Table 1 — Strains used for identification of O serogroup marker genes and tests on the algorithm in this study. Note:∗ GenBank (with prefix “GCA”) or CNGB (with prefix “CNA”) accession numbers for assembled genomes or sequences that harbor LPS gene clusters. [file Presentation_1.zip › Supplementary_tables/Table S8.docx]

**Table S8. Constitution of VPsero-predicted K serogroups that are inconsistent with serological determination.**

| **Transition pair**  **(Serology-VPsero)** | **VPsero-predicted K serogroup** | **Strain number** | **Strain numbers by K serogroup** |
| --- | --- | --- | --- |
| K13-K12 | K12 | 1 | 38 |
| KVIII-K12 | K12 | 1 |  |
| KII-K12 | K12 | 26 |  |
| KUK-K12 | K12 | 5 |  |
| KUT-K12 | K12 | 5 |  |
| K58-K6 | K6 | 1 | 12 |
| K6/K59-K6 | K6 | 2 |  |
| KUT-K6 | K6 | 9 |  |
| K41-K17 | K17 | 1 | 8 |
| Kuk-K17 | K17 | 6 |  |
| KUT-K17 | K17 | 1 |  |
| Kuk-K25 | K25 | 4 | 5 |
| KUT-K25 | K25 | 1 |  |
| Kuk-K23 | K23 | 5 | 5 |
| KIV-K56 | K56 | 1 | 3 |
| Kuk-K56 | K56 | 1 |  |
| KUT-K56 | K56 | 1 |  |
| KUK-K55 | K55 | 1 | 2 |
| KUT-K55 | K55 | 1 |  |
| KUT-K15 | K15 | 2 | 2 |
| KUT-K32 | K32 | 2 | 2 |
| KUT-K63 | K63 | 2 | 2 |
| KUT-K18 | K18 | 1 | 1 |
| Kuk-K30 | K30 | 1 | 1 |
| KUT-K42 | K42 | 1 | 1 |
| KUT-K70 | K70 | 1 | 1 |
| KUT-K9 | K9 | 1 | 1 |
| K10-Knt | Knt | 1 | 153 |
| K19-Knt | Knt | 1 |  |
| K20-Knt | Knt | 2 |  |
| K36-Knt | Knt | 2 |  |
| **K37-Knt** | Knt | 3 |  |
| **K53-Knt** | Knt | 1 |  |
| KVII-Knt | Knt | 2 |  |
| KVI-Knt | Knt | 1 |  |
| KUT-Knt | Knt | 94 |  |
| Kuk-Knt | Knt | 46 |  |
| Total |  | 237 | |

Note: 100 strains with unresolvable K serogroup information, namely KII\KIV\KVI\KVII\KVIII and Kuk/KUK.
